# Rationally designed HIV envelope glycoproteins delivered in a novel adjuvant elicited more broadly reactive antigen-specific antibody responses

**DOI:** 10.1186/1742-4690-9-S2-P18

**Published:** 2012-09-13

**Authors:** AK Dey, A Kassa, A Nandi, Y Sun, C Labranche, K Hartog, D Montefiori, A Carfi, I Srivastava, SW Barnett

**Affiliations:** 1 Novarits Vaccines & Diagnostics, Cambridge, MA, USA; 2 Department of Surgery, Duke University Medical Center, Durham, NC, USA

## Background

The identification of optimal antigen(s) and adjuvant combination(s) to elicit potent, protective, and long-lasting immunity has been a major challenge for the development of effective vaccines against HIV-1.

## Methods

Here, we designed disulfide-stabilized recombinant HIV-1 subtype B (SF162) envelope glycoproteins (Env), gp120 and gp140, by insertion of site-specific cysteine pairs between two layers (layer 1 and 2) in inner domain of gp120. In addition, we identified a novel adjuvant approach using Carbopol 971P, a cross-linked polyanionic carbomer, in combination with the Novartis proprietary oil-in water adjuvant, MF59, to augment humoral immune responses to the Env glycoprotein. We performed thorough in vitro analysis of the disulfide-stabilized Env glycoprotein followed by in vivo evaluations of the adjuvanted-Env glycoprotein boost in rabbits.

## Results

Intramuscular immunization of rabbits with disulfide-stabilized Env glycoproteins formulated in Carbopol 971P plus MF59 gave significantly higher titers of binding and virus neutralizing antibodies as compared to immunization using Env glycoprotein with either MF59 or Carbopol 971P alone. In addition, the antibodies generated were of higher avidity. Mapping of serum antibodies to determine epitope specificities showed that the disulfide-stabilized gp140 proteins elicited broader Env glycoprotein-specific antibody responses directed against epitopes that included the CD4-binding site, CD4-induced site and V1V2-loop. Importantly, the use of the novel adjuvant, Carbopol plus MF59, did not appear to present any obvious tolerability issues in animals upon intramuscular administration.

## Conclusion

Hence, the use of rationally stabilized Env-antigens in potent Carbopol 971P plus MF59 adjuvant may provide a benefit for evaluations of future vaccine against HIV-1.

